# Effects of Acute Beta-Alanine Ingestion and Immersion-Plus-Exercise on Connectedness to Nature and Perceived Pain

**DOI:** 10.3390/ijerph18158134

**Published:** 2021-07-31

**Authors:** R. W. Salatto, Graham R. McGinnis, Dustin W. Davis, Bryson Carrier, Jacob W. Manning, Mark DeBeliso, James W. Navalta

**Affiliations:** 1 Department of Kinesiology and Nutrition Science, University of Nevada, Las Vegas, NV 89154, USA; robert.salatto@unlv.edu (R.W.S.); graham.mcginnis@unlv.edu (G.R.M.); dustin.davis@unlv.edu (D.W.D.); bryson.carrier@unlv.edu (B.C.); 2 Department of Kinesiology and Outdoor Recreation, Southern Utah University, Cedar City, UT 84720, USA; jacobmanning@suu.edu (J.W.M.); markdebeliso@suu.edu (M.D.)

**Keywords:** green exercise, exercise perception, pain affect, hiking, supplementation

## Abstract

This double-blinded, placebo-controlled, crossover study examined the effect of induced painful sensation (via acute Beta Alanine (B-ALA) ingestion) on Love and Care of Nature (LCN), heart rate (HR), rating of perceived exertion (RPE), and McGill Pain Questionnaire (MPQ) during outdoor exercise. Twenty participants volunteered on consecutive days to complete a 0.8 km (0.5 mi) up-hill hike after consuming either B-ALA (6.4 g) or placebo. Immediately after consumption participants answered LCN, RPE, and MPQ questionnaires, immersed in a natural environment for 45 min, and then completed a hike as quickly as possible without running. No difference in HR (*p* = 0.846), or RPE (*p* = 0.606) were observed between treatments. Total MPQ scores increased with consumption of B-ALA (*p* = 0.001). An increased LCN score was observed following exercise regardless of condition (*p* = 0.035). The results demonstrate that acute B-ALA supplementation is effective in increasing perceived pain sensations. The results also demonstrate an increase in LCN in the presence of increased perceptions of pain sensations during exercise.

## 1. Introduction

The ability to connect with nature has several proposed benefits. These benefits include improved perceptual measures such as improved mood [1], decreased anxiety [2], decreased stress [3], and decreased depression [4]. Spending time in nature through green exercise (GE) has also led to increased social interactions [5], and increased desire to engage in outdoor exercise [6]. There are proposed physiologic benefits of natural engagement as well. Improvements in blood pressure [7], heart rate [4] blood glucose levels [8], and several markers of immune function [9].

Measurement tools attempt to evaluate one’s emotional relationship with the natural world, including the Connectedness to Nature Scale [10], and the Inclusion of Nature in Self [11]. Perkins [12] set out to develop a valid and reliable measurement tool for evaluating individuals’ love and affection for nature. The result of this endeavor produced the Love and Care of Nature scale (LCN), which demonstrated higher internal consistency (Cronbach’s α = 0.97) than previous tools while being reliable and valid for identifying one’s feelings for nature (*r* range = 0.69–0.91). To our knowledge, the LCN scale has not been investigated in conjunction with physical exercise.

Increasing evidence indicates that GE is beneficial to a number of psychological and physiological measures [13]. Green exercise is performed outdoors in natural environments, but do not necessarily need to be in settings with a preponderance of green space. A recent study found that individuals acclimated to a desert environment display similar beneficial responses in a natural desert environment as compared to a traditionally green setting [3]. The green exercise literature includes road cycling, mountain running, cross-country running, Boxercise, mountain biking, kayaking, and walking [2], mountain hiking [14], and solo-salsa dancing outdoors [15]. While studies have been conducted in various natural environments, to our knowledge, there are no investigations that have intentionally incorporated an acute nutritional supplement intervention.

Sports nutrition supplements such as protein and creatine [16], caffeine [17], and Beta Alanine (B-ALA) [18] in conjunction with exercise is a common occurrence. Beta alanine is a non-essential amino acid shown to increase muscle buffering capacity and decrease muscle fatigue [19]. B-ALA supplementation is an effective ergogenic aid when administered on a chronic basis over at least four weeks [20]. Acute B-ALA strategies have received less attention in the literature with findings being equivocal. One investigation reported no effect of acute B-ALA consumption on anaerobic capacity measures [21], while another found benefits on a limited time test at maximum aerobic speed [22]. One side effect of B-ALA ingestion is paresthesia, an unpleasant sensory symptom associated with heat and tingling [23]. Thus, B-ALA may be used to invoke sensory perception of painful sensations.

The human body experiences many subjective phenomena, two of which are pain and exertion. Pain is a perceptual response that has been defined as having both sensory and affective components [24]. The sensory component refers to the intensity of pain, and includes spatial (i.e., location in the body) and transitive qualifiers such as tingles and itches [25]. Pain affect refers to unpleasantness, and includes motivational aspects to seek protective action [26]. One of the most widely utilized tools to evaluate sensory and affective pain in clinical and research settings is the McGill Pain Questionnaire [27]. On the other hand, Borg’s perceived exertion scale (subjective measures from 6 to 20) during exercise is one of the most widely referenced tools in use [28]. The subjective perception of exertion is separate from fatigue, which is defined by a decrement in performance measures [29]. Perceived exertion has been found to be reduced during exercise in natural [30] or simulated natural environments [31]. While several studies have incorporated ratings of perceived exertion (RPE) measures into Beta alanine (B-ALA) supplementation trials [32], these have been with the intent of increasing performance rather than evaluating the effect of B-ALA on perceived exertion.

This study was designed to determine whether exercise performed in a natural environment would be impacted by the potential paresthesial side effects of a common nutritional supplement (B-ALA). While exercise increases the subjective feeling of exertion, this perception is reduced in a natural environment compared to bouts performed indoors [30]. Whereas B-ALA is a commonly utilized supplement to increase exercise performance (when taken chronically), it is unknown what effect it would have as an acute treatment on perceptual indices. The purpose of this study was to investigate the effects of acute B-ALA supplementation (as a means to increase perception of painful sensations) on hiking performance, connectedness to nature, perceptual responses, and performance measures. We hypothesized that B-ALA supplementation would increase the perception of pain, and that exercise would increase connectedness to nature.

## 2. Materials and Methods

### 2.1. Participants

The study was designed as a double-blind, placebo-controlled crossover investigation. Participants completed Institutional Review Board approved informed consent forms (approval #01-102019a, #1508568) and the American College of Sports Medicine (ACSM) Health Risk Questionnaire before participating [33]. Participants were eligible to participate if it was deemed that medical clearance was not necessary according to the ACSM algorithm (i.e., no cardiovascular, metabolic or renal disease and no signs or symptoms suggestive of these diseases). Twenty participants (*n* = 4 females, *n* =16 males) were recruited through word of mouth with the following demographic data (reported as *M* ± *SD*): age = 24 ± 7 years, height = 177.26 ± 8.83 cm, body mass = 78.26 ± 14.55 kg.

### 2.2. Protocol

Participants reported on two consecutive days in the months of October and November 2020 to the Thunderbird Trailhead in Cedar City, Utah. Each visit, participants were fitted with Polar heart rate monitors (Polar T31 heart rate monitor, Polar Electro Inc., Bethpage, NY, USA). The mechanism of B-ALA (CarnoSyn, NOW Foods, Bloomingdale, IL, USA) or placebo (PLA, caffeine-free sugar-free Crystal Light) administration in an applied setting and randomization procedure has been described previously [34]. Participants were not given any information about potential benefits or side effects of the liquid they consumed. Participants also were blind to the potential of paresthesia, specifically, as a possible side effects of B-ALA ingestion. Briefly, participants consumed one of two treatments in a counterbalanced order between experimental days: 6.4 g B-ALA or 2 g Crystal Light each dissolved in ~230 mL water with both treatments being identical in color, taste, smell, and texture.

Immediately after consumption, participants answered several questionnaires that served as the baseline measurement for primary outcomes: the Love and Care of Nature (LCN, fifteen questions with seven Likert-type answers ranging from Very strongly disagree to Very strongly agree) [12], Borg Rating of Perceived Exertion (6–20 scale) (RPE) [35], and McGill Pain Questionnaire (MPQ, fifteen sensations rated as None, Mild, Moderate, or Severe) short form [36]. Heart rate was measured as a secondary outcome. Participants were directed to locate a comfortable spot where they could sit alone quietly with no electronic devices and immerse themselves in the natural environment for the ensuing 45 min. The trail is situated at an initial elevation of 1775 km (5,825 feet), with an environment including Pinion Pine and Juniper trees interspersed with low lying plants and shrubbery. Additionally, the landscape contained rocks in shades of red, orange, and brown, consistent with what is found in the mountainous regions of Southern Utah. Mean environmental conditions across testing sessions were as follows: temperature = 286.9 ± 261.7 K (13.8 ± 11.5 °C, 56.8 ± 11.3 °F), wind speed = 1.9 ± 1.2 m·s^−1^ (4.3 ± 2.6 mph), relative humidity = 15.3 ± 4.9%.

**Figure 1:**
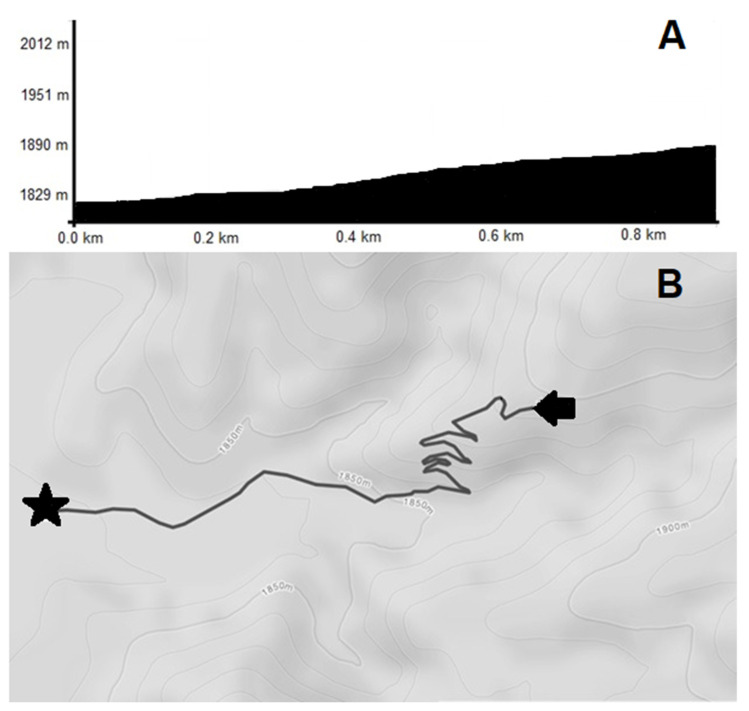
(**A**) Elevation gain experienced by participants over the 0.8 km (0.5 mi) hike. (**B**) Terrain profile of the Lightning Switch Trail section completed by participants.

After immersion, participants completed a self-paced 0.8 km (0.5 mi) uphill hike (66 m [217 ft] elevation change,) on the Lightning Switch trail in Cedar City, UT, USA, (see Figure 1) as fast as possible without running. Perceived pain (MPQ), LCN, RPE, HR and exercise time (time being a secondary outcome) were noted immediately upon completion of the hike. To minimize the possibility of delayed-onset muscle soreness from hiking downhill between testing sessions, participants were instructed to return to the trailhead in a slow controlled manner.

In order to preserve the nature of the blind, participants, throughout the entirety of the study, were given strict instructions not to discuss any of their experiences with any other participants, or with researchers. One member of the research staff was selected to serve as the blind/randomization facilitator as well as a “safety officer”. Participants were told that if they had any questions or concerns, they could speak to the “safety officer” and no one else. This member of the research team was instructed not to share participants’ comments/concerns with any other participant or researchers unless in the case of emergency. 

### 2.3. Statistical Analysis

The dependent variables of total pain, sensory pain, pain affect, and ratings of perceived exertion were analyzed using a 2 (Treatment: B-ALA, PLA) × 2 (Exercise time: pre, post) repeated measures ANOVA. A 2 (condition: PL, B-ALA) × 2 (time: Day 1, Day 2) repeated measures ANOVA was run for the dependent variables of hike time, HR, and LCN. These repeated measures ANOVAs, where necessary, were followed by post hoc paired-samples *t*-tests with Bonferroni corrections. Significance was accepted at *p* < 0.05. The relationship between perceived pain measures and perceived exertion was evaluated using Pearson Product Moment Correlation Coefficients. Data were analyzed using the IBM SPSS Statistics software package (Version 26, IBM Corporation, Armonk, NY, USA).

## 3. Results

### 3.1. Performance Variables

Hiking performance was not different between supplementation treatments (PLA = 8.6 ± 2.0 min, B-ALA = 8.5 ± 2.3 min, *p* = 0.78, r = 0.937). There was no treatment × time interaction for HR (*p* = 0.897, η_p_^2^ = 0.001) or main effect for treatment (*p* = 0.846, η_p_^2^ = 0.002). There was a main effect for time on HR (pre = 77 ± 3 bpm, post = 152 ± 4 bpm, *p* > 0.001, η_p_^2^ = 0.950). 

### 3.2. Love and Care of Nature

**Figure 2:**
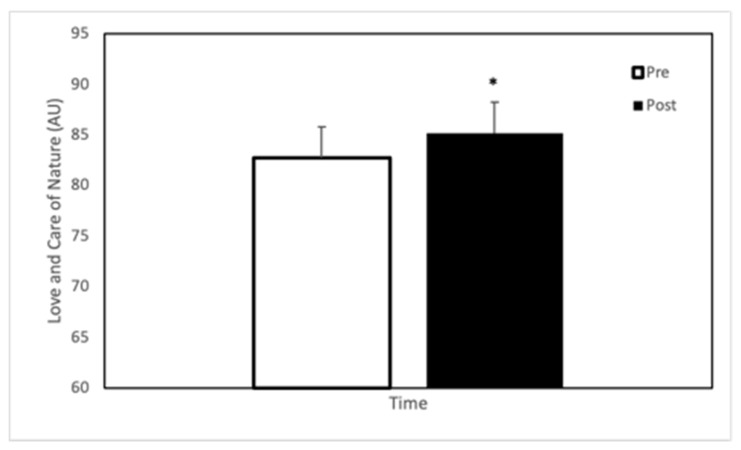
Love and Care of Nature scale (expressed as arbitrary units) in participants (*n* = 20) at baseline and following a 0.8 km (0.5 mi) hike. * represents significant difference (*p* < 0.05).

There was no interaction between treatment and time on LCN (*p* = 0.330, PES = 0.05). There was no main effect of treatment on LCN (*p* = 0.863, η_p_^2^ = 0.002). There was a main effect for time on LCN score (see Figure 2 *p* = 0.035, η_p_^2^ = 0.214), with participants increasing emotional connectedness to nature following exercise (see Figure 2). 

### 3.3. Perceptual Variables

**Figure 3:**
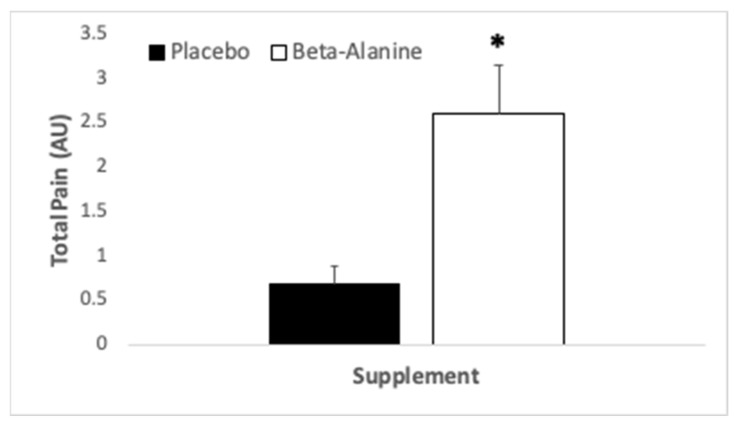
Perception of total pain (arbitrary units), as determined by the McGill Pain Questionnaire short form, in participants (*N* = 20) who consumed β-alanine prior to immersion in nature and a subsequent hike. * indicates significant difference between supplementation trials.

No interaction (*p* = 0.29, η_p_^2^ = 0.06) or main effect of exercise (*p* = 0.17, η_p_^2^ = 0.10) was noted for total pain score, but a main effect of treatment was observed (*p* = 0.001, η_p_^2^ = 0.46). Participants perceived greater total pain over the course of the B-ALA supplementation trial when compared to PLA (see Figure 3). 

**Figure 4:**
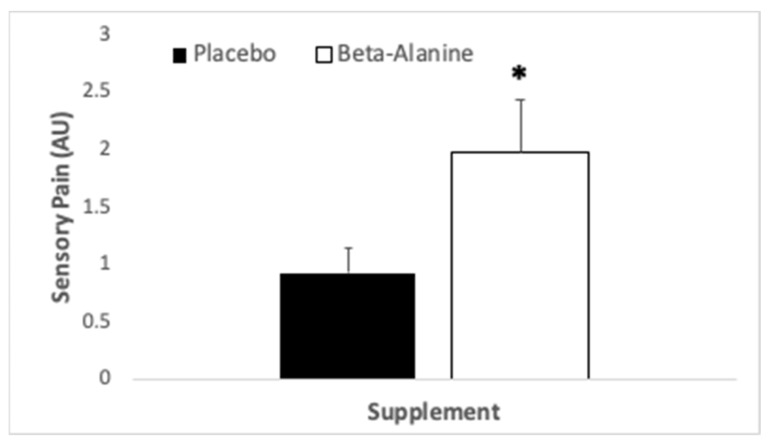
Sensory pain (arbitrary units), as perceived by participants (*N* = 20) who consumed β-alanine prior to immersion in nature and a subsequent hike. * indicates significant difference between trials.

No interaction was present for the sensory component of pain (*p* = 0.15, η_p_^2^ = 0.11), nor for the main effect of exercise (*p* = 0.12, η_p_^2^ = 0.12), but a main effect for treatment was observed (*p* = 0.006, η_p_^2^ = 0.34). Participants perceived increased sensory pain with B-ALA supplementation than PLA (see Figure 4).

**Figure 5:**
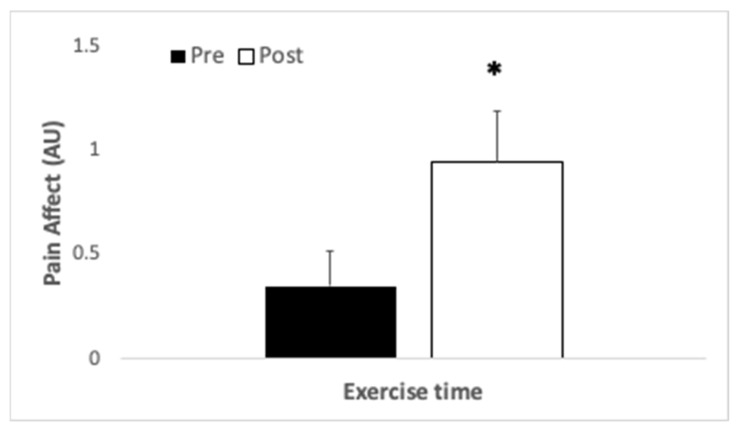
Pain affect (arbitrary units), perceived by participants (*N* = 20) who completed an uphill hike as quickly as possible after consumption of β-alanine or placebo prior to immersion in nature. * indicates significant difference between times sampled.

When the affective component of pain was considered, no interaction (*p* = 1.0, η_p_^2^ < 0.001) or main effect for treatment was found (*p* = 1.0, η_p_^2^ < 0.001), but a main effect for exercise time was observed (*p* = 0.05, η_p_^2^ = 0.19). Participants perceived greater pain affect upon completion of exercise compared with baseline (see Figure 5).

**Figure 6:**
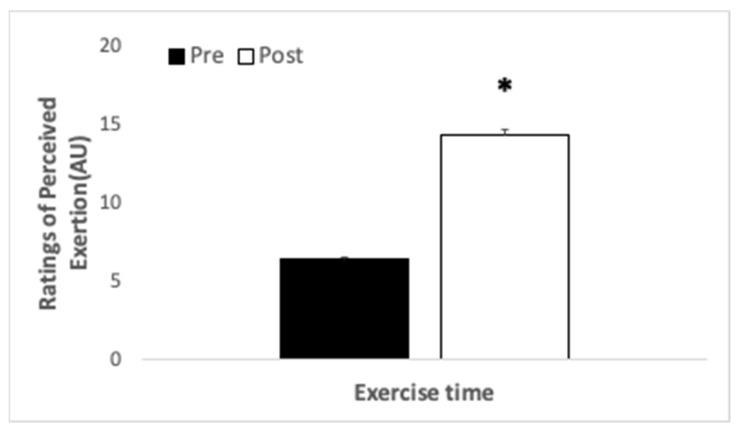
Ratings of perceived exertion (arbitrary units), in participants (*N* = 20) who completed a hike as quickly as possible after β-alanine or placebo consumption. * indicates significant difference between times sampled.

No interaction was observed for subjective ratings of perceived exertion (*p* = 1.0, η_p_^2^ < 0.001) or main effect of supplementation treatment (*p* = 0.606, η_p_^2^ = 0.014). A main effect of exercise time on RPE was observed (*p* < 0.001, η_p_^2^ 0.947). Participants perceived increased exertion upon completion of the uphill hike compared with baseline (see Figure 6). RPE was correlated with total pain (r = 0.4293, r^2^ = 0.1843, *p* < 0.001), sensory pain (r = 0.3823, r^2^ = 0.1462, *p* < 0.001), and pain affect (r = 0.3804, r^2^ = 0.1447, *p* < 0.001).

## 4. Discussion

The purpose was to evaluate perceptual measures during outdoor activity under the combination of supplementation-induced painful sensations and exercise. The main findings are that based on increased post-exercise Love and Care of Nature scores, participants’ connectedness to nature increased with exercise. The results also demonstrate that acute B-ALA supplementation has no negative effects on participants’ connectedness to nature. This is impactful given that total pain and sensory pain were influenced by B-ALA consumption, independent of exercise. Interestingly, pain affect, and ratings of perceived exertion were influenced by exercise, independent of B-ALA supplementation. We found that each measure of pain was correlated with RPE.

Green exercise has been shown to positively impact mental and physical health. Mackay et al. [2] demonstrated that outdoor exercise reduced state anxiety with the greatest reductions coming from road cycling, mountain biking, and Boxercise. Niedermeier et al. [14] demonstrated mountain hiking lead to decreased salivary cortisol levels compared to a sedentary control condition. Pretty et al. [37] demonstrated that exposure to scenes of peaceful green environments lead to improved affect compared to those of urban environments. While much research is available to demonstrate the positive effects of GE, we report for the first time that LCN increased with exercise in an outdoor environment (increased with GE). While much research is available to demonstrate the positive impacts of GE, at the time of submission, there is no reported research investigating the effects of GE on the Love and Care of Nature scale. A novel finding, reported for the first time, is that green exercise led to an increased connectedness to nature as evidenced by increased LCN scores post exercise. 

This increased love and connectedness to nature may be a critical factor in the future desire of individuals to seek out natural environments and to engage in recreational activities (i.e., green exercise). The pattern of engagement with nature, and exercise in nature is cyclical in essence. The engagement in GE will likely improve mental and emotional health, and now, as evidenced by the findings of the current study, connectedness to nature. Those who spend time exercising in natural environments may develop a deeper connection to, and love of nature, and have a stronger desire for subsequent bouts of GE. Subsequent bouts of GE may lead to chronic improvements in mental and physical health. Future investigations to test this interaction are warranted.

To our knowledge, this is the first investigation to report the effect of B-ALA supplementation on total pain score as measured by the MPQ. It was found that 6.4 g of B-ALA was sufficient to significantly increase the perception of total pain in participants, and that an exercise bout did not influence this sensation. Clinical investigations have reported a decrease in total pain following the administration of supplements including calcium fructoborate in individuals with knee discomfort [38], and duloxetine in patients with peripheral neuropathy caused by diabetes [39]. The current investigation is different in that the supplement increased the perception of total pain. A limited number of investigations have reported that nutritional supplements are capable of inducing the perception of pain including niacin (increased dental pain) [40], and glyceryltrinitrate (migraine and cluster headache) [41]. While the exact mechanism of paresthesia from B-ALA supplementation is unknown [42] it has been suggested to excite nociceptive sensory receptors located on the dorsal root ganglion [43]. It should be noted that in the current study, B-ALA increased the perception of total pain, and this was independent of the effect of exercise in nature. Taken another way, the exercise employed in the current investigation did not contribute to the total pain perceived by participants.

The sensory component of pain signifies the perceived intensity, and includes characteristics such as tingles and itches [25]. Since one common side effect of B-ALA consumption is paresthesia [23], it is not surprising that supplementation increased perception of the sensory component of pain in the current study. One other study has utilized β-alanine supplementation and the McGill Pain Questionnaire sensory dimension [42]. Décombaz et al. studied the effect of a slow-release B-ALA tablet compared to delivery in an aqueous solution, and while no difference in the sensory perception of pain was observed between treatments, an increase in pain intensity measured by other pain scales was reported [42]. Other nutritional supplements have been associated with sensory characteristics of pain including Vitamin B6 (sensory neuronal pain) [44], and folate (high intake being associated with peripheral neuropathy in individuals with a specific gene variant) [45]. Additionally, one investigation utilizing the opioid receptor blocker naloxone in patients undergoing knee arthroscopy found no differences between total pain score and the sensory component [46]. The results of the current study are similar in that B-ALA increased total pain as well as the sensory component, independent of the influence of exercise. 

The affective component of pain represents a rating of pain unpleasantness and is known to increase as the intensity of the stimulus increases [26]. In the current study, the perception of the affective component of pain was not influenced by supplementation but increased after the exercise bout. In this context, the intensity of exercise as a stimulus was greatly increased, as participants transitioned from a seated period immersed in nature to hiking as quickly as possible on a vertical incline. Thus, it could be expected that the perception of pain affect would increase. Furthermore, the predictability of a painful stimulus is known to result in lower pain affect compared to a stimulus that is unpredictable [47]. While the participants were not polled with respect to which ones were familiar with the hiking trail utilized in the current study, it was anecdotally noted that very few were aware of the locale of the trailhead based on directions that were provided and further communication guiding participants to the study site. Given this, another potential explanation for the increase in pain affect noted is unfamiliarity with what the hiking task would involve. Future investigations evaluating the effect of familiarity of exercise in a natural environment on pain affect are important to further determine the underlying mechanism associated with the phenomenon observed in the current study.

Ratings of perceived exertion is a commonly used adjunct to physiological data collected during exercise investigations. RPE is known to increase significantly when participants hike on an incline as high as 17.6% [48], and the current investigation provides further evidence toward this end. B-ALA supplementation did not influence RPE in participants who performed repeated upper body Wingate tests [49], overall loads in participants who completed 4 weeks of an 800 m training regimen [50], nor untrained individuals who performed in a lower body resistance training session [51]. The current results provide further evidence that B-ALA supplementation would not be expected to affect RPE, extending our observation to a common green exercise modality (hiking). In the present investigation, RPE was significantly correlated with all measures of pain from the McGill Pain Questionnaire. These results are in line with others who have observed pain ratings to correlate with exertion during a symptom-limited graded exercise test [52], and while participants performed increasing workloads on a cycle ergometer [53].The results of this current study demonstrate that acute supplementation with B-ALA had no effect on hiking performance. These results are in agreement with Glenn et al. [21] who demonstrated no differences in performance during repeated Wingate cycling tests between B-ALA and control in female cyclists acutely supplementing B-ALA. The current results also agree with Invernizzi et al. [54] who found no performance differences in Run-Based Anaerobic Sprint Tests (RAST) in recreationally active men acutely supplementing B-ALA. In the current study, no difference in RPE was observed between B-ALA and placebo. These results demonstrate that acute supplementation with B-ALA had no negative effects on perceived exertion. This is in agreement with the findings of Invernizzi et al. [54] and Gross et al. [55]. 

For the current study, no significant difference in HR was observed between treatments. This is in contrast to the results of Gross et al. [55] who found that participants in a placebo group had higher peak heart rates and spent more time with their heart rates above 90% of peak compared to those who acutely ingested B-ALA when completing a high intensity interval training workout. This discrepancy suggests that B-ALA may have different effects on HR depending on exercise modality or intensity. Comparisons between the current study and Gross et al. may be difficult because of the extreme differences in supplementation duration. Due to a paucity of literature investigating the effects of B-ALA on HR during exercise, future research is needed to draw definitive conclusions. 

The current study is not without limitations. It is known that among individuals of Asian descent, females experience paresthesia to a greater extent than males following ingestion of B-ALA [56]. Ethnicity was not controlled for in the current investigation, and future studies would be advised to collect and report this demographic data. The current investigation employed an acute B-ALA supplementation strategy with 24 h between trials. Previous research has suggested a 5–15 week wash out period for chronic B-ALA administration [57], and thus it is possible that residual effects could have influenced participants who received B-ALA on the first trial day. As the B-ALA half-life is 25 min [23] and as there was no difference in the performance measure of hiking time, we do not believe there was a prolonged effect of B-ALA in those participants. Green exercise is known to decrease perceptions of exertion associated with exercise [30]. It is unknown whether a blunting effect on the perceptual measures of the current study occurred because hiking employed was in a natural environment. Future studies should incorporate an indoor or urban component to determine the potential influence of this effect.

## 5. Conclusions

Visiting nature and exercising in outdoor settings are known to be beneficial. The results of this study indicate that one’s perception of love and care of nature is increased when exposure to nature is carried out in conjunction with physical exercise. Furthermore, the potential effects of supplementation during green exercise are unknown. Beta alanine is known to induce a tingling sensation (paresthesia) in users. This sensation can be unpleasant for some. Acute β-alanine ingestion significantly increased both total pain and the sensory component of pain, while having no effect on pain-affect, hike performance, heart rate, or ratings of perceived exertion. Love and care of nature increased post-exercise despite B-ALA-supplementation-induced pain therefore increases in painful sensations do not necessarily negate one’s connectedness to the natural world.

## Data Availability

The data included in this study are available on request from the corresponding author.

## References

[1] Barton J., Griffin M., Pretty J. (2012). Exercise-, nature-and socially interactive-based initiatives improve mood and self-esteem in the clinical population. Perspect. Public Health.

[2] Mackay G.J., Neill J.T. (2010). The effect of “green exercise” on state anxiety and the role of exercise duration, intensity, and greenness: A quasi-experimental study. Psychol. Sport Exerc..

[3] Navalta J.W., Bodell N.G., Tanner E.A., Aguilar C.D., Radzak K.N. (2021). Effect of exercise in a desert environment on physiological and subjective measures. Int. J. Environ. Health Res..

[4] Han J.-W., Choi H., Jeon Y.-H., Yoon C.-H., Woo J.-M., Kim W. (2016). The effects of forest therapy on coping with chronic widespread pain: Physiological and psychological differences between participants in a forest therapy program and a control group. Int. J. Environ. Res. Public Health.

[5] Rogerson M., Gladwell V., Gallagher D., Barton J. (2016). Influences of green outdoors versus indoors environmental settings on psychological and social outcomes of controlled exercise. Int. J. Environ. Res. Public Health.

[6] Focht B.C. (2009). Brief walks in outdoor and laboratory environments: Effects on affective responses, enjoyment, and intentions to walk for exercise. Res. Q. Exerc. Sport.

[7] Duncan M., Clarke N., Birch S., Tallis J., Hankey J., Bryant E., Eyre E. (2014). The effect of green exercise on blood pressure, heart rate and mood state in primary school children. Int. J. Environ. Res. Public Health.

[8] Ohtsuka Y., Yabunaka N., Takayama S. (1998). Shinrin-yoku (forest-air bathing and walking) effectively decreases blood glucose levels in diabetic patients. Int. J. Biometeorol..

[9] Mao G.X., Lan X.G., Cao Y.B., Chen Z.M., He Z.H., Lv Y.D., Wang Y.Z., Hu X.L., Wang G.F., Jing Y. (2012). Effects of short-term forest bathing on human health in a broad-leaved evergreen forest in Zhejiang Province, China. Biomed. Environ. Sci..

[10] Mayer F.S., Frantz C.M. (2004). The connectedness to nature scale: A measure of individuals’ feeling in community with nature. J. Environ. Psychol..

[11] Martin C., Czellar S. (2016). The extended inclusion of nature in self scale. J. Environ. Psychol..

[12] Perkins H.E. (2010). Measuring love and care for nature. J. Environ. Psychol..

[13] Gladwell V.F., Brown D.K., Wood C., Sandercock G.R., Barton J.L. (2013). The great outdoors: How a green exercise environment can benefit all. Extrem. Physiol. Med..

[14] Niedermeier M., Grafetstätter C., Hartl A., Kopp M. (2017). A randomized crossover trial on acute stress-related physiological responses to mountain hiking. Int. J. Environ. Res. Public Health.

[15] Byrka K., Ryczko N. (2018). Positive effects of dancing in natural versus indoor settings: The mediating role of engagement in physical activity. J. Environ. Psychol..

[16] Bemben M., Witten M., Carter J., Eliot K., Knehans A., Bemben D. (2010). The effects of supplementation with creatine and protein on muscle strength following a traditional resistance training program in middle-aged and older men. J. Nutr. Health Aging.

[17] Salatto R.W., Arevalo J.A., Brown L.E., Wiersma L.D., Coburn J.W. (2018). Caffeine’s Effects on an Upper-Body Resistance Exercise Workout. J. Strength Cond. Res..

[18] Kendrick I.P., Harris R.C., Kim H.J., Kim C.K., Dang V.H., Lam T.Q., Bui T.T., Smith M., Wise J.A. (2008). The effects of 10 weeks of resistance training combined with β-alanine supplementation on whole body strength, force production, muscular endurance and body composition. Amino Acids.

[19] Stout J.R., Cramer J.T., Zoeller R.F., Torok D., Costa P., Hoffman J.R., Harris R.C., O’Kroy J. (2007). Effects of beta-alanine supplementation on the onset of neuromuscular fatigue and ventilatory threshold in women. Amino Acids.

[20] Stout J.R., Cramer J.T., Mielke M., O’Kroy J., Torok D.J., Zoeller R.F. (2006). Effects of twenty-eight days of beta-alanine and creatine monohydrate supplementation on the physical working capacity at neuromuscular fatigue threshold. J. Strength Cond. Res..

[21] Glenn J.M., Smith K., Moyen N.E., Binns A., Gray M. (2015). Effects of Acute Beta-Alanine Supplementation on Anaerobic Performance in Trained Female Cyclists. J. Nutr. Sci. Vitaminol..

[22] Huerta-Ojeda A., Contreras-Montilla O., Galdames-Maliqueo S., Jorquera-Aguilera C., Fuentes-Kloss R., Guisado-Barrilao R. (2019). Effects of acute supplementation with beta-alanine on a limited time test at maximum aerobic speed on endurance athletes. Nutr. Hosp..

[23] Harris R.C., Tallon M.J., Dunnett M., Boobis L., Coakley J., Kim H.J., Fallowfield J.L., Hill C.A., Sale C., Wise J.A. (2006). The absorption of orally supplied beta-alanine and its effect on muscle carnosine synthesis in human vastus lateralis. Amino Acids.

[24] Talbot K., Madden V.J., Jones S.L., Moseley G.L. (2019). The sensory and affective components of pain: Are they differentially modifiable dimensions or inseparable aspects of a unitary experience? A systematic review. Br. J. Anaesthiol..

[25] Auvray M., Myin E., Spence C. (2010). The sensory-discriminative and affective-motivational aspects of pain. Neurosci. Biobehav. Rev..

[26] Price D.D. (2000). Psychological and neural mechanisms of the affective dimension of pain. Science.

[27] Melzack R., Katz J. (2001). The McGill Pain Questionnaire: Appraisal and current status. Handbook of Pain Assessment.

[28] Borg G.A. (1970). Perceived exertion as an indicator of somatic stress. Scand. J. Rehabil. Med..

[29] Watt B., Grove R. (1993). Perceived exertion. Antecedents and applications. Sports Med..

[30] Calogiuri G., Litleskare S., Fagerheim K.A., Rydgren T.L., Brambilla E., Thurston M. (2017). Experiencing Nature through Immersive Virtual Environments: Environmental Perceptions, Physical Engagement, and Affective Responses during a Simulated Nature Walk. Front. Psychol..

[31] Akers A., Barton J., Cossey R., Gainsford P., Griffin M., Micklewright D. (2012). Visual color perception in green exercise: Positive effects on mood and perceived exertion. Environ. Sci. Technol..

[32] Smith A.E., Stout J.R., Kendall K.L., Fukuda D.H., Cramer J.T. (2012). Exercise-induced oxidative stress: The effects of beta-alanine supplementation in women. Amino Acids.

[33] Medicine, American College of Sports (2013). ACSM’s Guidelines for Exercise Testing and Prescription.

[34] Salatto R.W., Davis D.W., Carrier B., Barrios B., Sertic J.V.L., Cater P.C., Navalta J.W. (2020). Efficient method of delivery for powdered supplement or placebo for an outdoor exercise investigation. Top. Exerc. Sci. Kinesiol..

[35] Borg G.A. (1982). Psychophysical bases of perceived exertion. Med. Sci. Sports Exerc..

[36] Melzack R. (1987). The short-form McGill Pain Questionnaire. Pain.

[37] Pretty J.N., Griffin M., Peacock J., Hine R., Sellens M., South N. (2005). A Countryside for Health and Wellbeing: The Physical and Mental Health Benefits of Green Exercise.

[38] Pietrzkowski Z., Mercado-Sesma A.R., Argumedo R., Cervantes M., Nemzer B., Reyes-Izquierdo T. (2018). Effects of once-daily versus twice daily dosing of calcium fructoborate on knee discomfort. A 90 day, double-blind, placebo controlled randomized clinical study. J. Aging Res. Clin. Pract..

[39] Raskin J., Pritchett Y.L., Wang F., D’Souza D.N., Waninger A.L., Iyengar S., Wernicke J.F. (2005). A double-blind, randomized multicenter trial comparing duloxetine with placebo in the management of diabetic peripheral neuropathic pain. Pain Med..

[40] Leighton R.F., Gordon N.F., Small G.S., Davis W.J., Ward E.S. (1998). Dental and gingival pain as side effects of niacin therapy. Chest.

[41] Costa A., Ravaglia S., Sances G., Antonaci F., Pucci E., Nappi G. (2003). Nitric oxide pathway and response to nitroglycerin in cluster headache patients: Plasma nitrite and citrulline levels. Cephalalgia.

[42] Decombaz J., Beaumont M., Vuichoud J., Bouisset F., Stellingwerff T. (2012). Effect of slow-release beta-alanine tablets on absorption kinetics and paresthesia. Amino Acids.

[43] Liu Q., Sikand P., Ma C., Tang Z., Han L., Li Z., Sun S., LaMotte R.H., Dong X. (2012). Mechanisms of itch evoked by beta-alanine. J. Neurosci..

[44] Vrolijk M.F., Opperhuizen A., Jansen E., Hageman G.J., Bast A., Haenen G. (2017). The vitamin B6 paradox: Supplementation with high concentrations of pyridoxine leads to decreased vitamin B6 function. Toxicol Vitr..

[45] Sawaengsri H., Bergethon P.R., Qiu W.Q., Scott T.M., Jacques P.F., Selhub J., Paul L. (2016). Transcobalamin 776C-->G polymorphism is associated with peripheral neuropathy in elderly individuals with high folate intake. Am. J. Clin. Nutr..

[46] Stein C., Hassan A.H., Lehrberger K., Giefing J., Yassouridis A. (1993). Local analgesic effect of endogenous opioid peptides. Lancet.

[47] Carlsson K., Andersson J., Petrovic P., Petersson K.M., Ohman A., Ingvar M. (2006). Predictability modulates the affective and sensory-discriminative neural processing of pain. Neuroimage.

[48] Montes J., Stone T.M., Manning J.W., McCune D., Tacad D.K., Young J.C., Debeliso M., Navalta J.W. (2015). Using Hexoskin Wearable Technology to Obtain Body Metrics During Trail Hiking. Int. J. Exerc. Sci..

[49] Tobias G., Benatti F.B., de Salles Painelli V., Roschel H., Gualano B., Sale C., Harris R.C., Lancha A.H., Artioli G.G. (2013). Additive effects of beta-alanine and sodium bicarbonate on upper-body intermittent performance. Amino Acids.

[50] Ducker K.J., Dawson B., Wallman K.E. (2013). Effect of beta-alanine supplementation on 800-m running performance. Int. J. Sport Nutr. Exerc. Metab..

[51] Roveratti M.C., Jacinto J.L., Oliveira D.B., da Silva R.A., Andraus R.A.C., de Oliveira E.P., Ribeiro A.S., Aguiar A.F. (2019). Effects of beta-alanine supplementation on muscle function during recovery from resistance exercise in young adults. Amino Acids.

[52] Borg G., Ljunggren G., Ceci R. (1985). The increase of perceived exertion, aches and pain in the legs, heart rate and blood lactate during exercise on a bicycle ergometer. Eur. J. Appl. Physiol. Occup. Physiol..

[53] Cook D.B., O’Connor P.J., Eubanks S.A., Smith J.C., Lee M. (1997). Naturally occurring muscle pain during exercise: Assessment and experimental evidence. Med. Sci. Sports Exerc..

[54] Invernizzi P.L., Benedini S., Saronni S., Merati G., Bosio A. (2013). The acute administration of Carnosine and beta-Alanine does not improve running anaerobic performance and has no effect on the metabolic response to exercise. Adv. Phys. Educ..

[55] Gross M., Boesch C., Bolliger C.S., Norman B., Gustafsson T., Hoppeler H., Vogt M. (2014). Effects of beta-alanine supplementation and interval training on physiological determinants of severe exercise performance. Eur. J. Appl. Physiol..

[56] Macphee S., Weaver I.N., Weaver D.F. (2013). An Evaluation of Interindividual Responses to the Orally Administered Neurotransmitter beta -Alanine. J. Amino Acids.

[57] Baguet A., Reyngoudt H., Pottier A., Everaert I., Callens S., Achten E., Derave W. (2009). Carnosine loading and washout in human skeletal muscles. J. Appl. Physiol..

